# Report of a Case of Inversio-Uteri, of Two Years Standing. Reduced by Taxis

**Published:** 1875-04

**Authors:** B. F. Dawson


### Report of a Case of Inversio-Uteri of Two Years' Standing, Reduced by Taxis; with Remarks.

BY B. F. DAWSON, M. D.

On the 6th of January last, Mrs. R. was sent to me by Dr. Jeffrey Bourke, of this city, with the view of confirming his diagnosis of her case, and for advice as to her treatment. The patient was a naturally strong woman, aged thirty-eight years, and the mother of five children. Her illness dated from the birth of her last child, two years ago, of which the following is the account, as given by herself and husband :

On February 5, 1873 (two years ago), she was taken in labor with her fifth child, which was born in four hours without assistance or trouble. In delivering the after-birth, however, her physician caused her considerable pain by pulling upon the cord, of which she complained, and when he persisted she cried out to him repeatedly to stop ; at the same time she was losing blood freely. She soon lost consciousness, and her husband and family thought her to be dying. For several days after she was so prostated that she was not expected to recover, even by her physicians. She continued to lose blood freely, and a large mass or tumor protruded from the vulva, requiring repeated efforts at reduction. Her water, also, had to be drawn off for a couple of weeks. In about three weeks after the birth of her child she began to improve, losing less blood, and having less pain. In the mean time she had used vaginal washes and injections ordered by her physicians, and which seemed to control the bleeding considerably. Thus matter remained for about a year, with, however. a yellowish discharge throughout, and occasionally clots of blood. The protruding tumor also gave her considerable trouble, coming down repeatedly on the slightest effort, and requiring her to lie down and have it replaced by her husband. In February, 1874, a year after the birth of her child, the hæmorrhage returned with great severity, and continued without abatement until April, when it intermitted for a few days ; again recurring, however, profusely for several weeks. The patient in consequence became rapidly exhausted to such a degree as to be unable to raise her head off her pillow. Her physicians again attempted to control the hæmorrhage by injection and tamponing the vagina with cloths, but to no avail. Her condition continued thus until last November (1874), when she was seen by Dr. Jeffrey Bourke, of this city. After an examination of her, he expressed to her physicians his opinion that she was undoubtedly suffering from inversion ; and again in December he adhered to the same opinion, and advised a consultation with others for treatment. With this object he consulted me about her, and at my suggestion and advice she came to this city. From the time we saw her in November to the date of my first visit she continued to lose blood, but had gained somewhat in strength. As already stated, she was seen by me January 6, 1875, and on careful examination I found her suffering from complete inversio-uteri. The uterus occupied the entire vagina, was firm to the touch, bled readily, and was exceedingly sensitive. The ring formed by the vaginal portion of the cervix was thin, and did not constrict the tumor to any great degree. In all respects the case seemed one not likely to afford great trouble at reduction, and accordingly I felt safe in expressing my opinion to the patient and her husband that she could be cured if she would submit to treatment. The diagnosis and above opinions were concurred in by my friend Dr. Munde, who saw her at the same time with me. After some persuasion she consented to place herself under my treatment, notwithstanding her friends urged her strongly not to risk her life by an operation. The following day, at my request, my friend Prof. T. G. Thomas examined the case, and expressed the opinion that it was a case favorable to success in all respects, notwithstanding the existence of the inversion for so long a time as two years.

On January 13th, the patient being etherized, and having used hot vaginal injections for the previous week, I made my attempt. My efforts I had determined to confine wholly to taxis, one hand in the vagina embracing the tumor within the fingers, and thus forcing them up within the ring, dilating the latter and carrying the *cervix uteri* up, at the same time making counter-pressure through the abdominal walls, as a centre to press against as well as to guard against rupture of the vagina. In these efforts I was relieved when tired by Drs. Bourke and Henry Nicoll. After two and a half hours no success of any amount attended our efforts, and accordingly the patient was replaced in bed, it being decided best not to subject her too long to the influence of anæsthasia, or the uterus to prolonged manipulation.

On January 16th, three days after, I prepared for a second attempt, but, after manipulating for two hours, the uterus became œdematous, and, with the concurrence of Drs. Nicoll and J C. Jay, Jr., it was deemed best to desist, and give the patient a few days' rest, and subject the parts to the influence of a constant use of hot vaginal douches. After both these attempts there was a slight elevation of temperature, some pain, very slight discharge, and no vomiting. By the use of quinine and morphia the fever and pain were controlled, and nourishing diet kept up the patient's strength.

On January 18th, I essayed a third attempt, but soon found it would be to my advantage, as well as to the patient's, to subject the uterus still longer to the action of hot water, as it was still coriaceous and doughy in feeling. A full week was given the patient, and on January 25th, efforts were again renewed, in like manner as before, excepting that I essayed the use of Dr. White's inversion repositor, but laid it aside for reasons to be hereafter stated. From the outset it seemed that success would crown our efforts at last, as the ring was largely dilated and dilatable, the utetus soft and pliable, and the fundus, after a slight effort, could be carried up to the ring. Through the abdominal walls the enlarged ring was easily felt, and the finger could be forced into the depression of the inversion. Alternately relieving one another, after one hour and twenty minutes, the fundus was well up within the ring. I was on the point of again resting, when suddenly the left horn of the uterus yielded to the pressure of the thumb of my right hand. Following up this ground gained, I succeeded, in a few minutes, in completely replacing the inversion, and felt the uterus contract considerably upon my finger in its cavity. The patient being then examined by Drs. Nicoll and Bourke at my request, she was replaced in bed, hot cloths were applied over the abdomen, and, after her recovery out of the anæsthetic, quinine and morphia controlled the fever and pain. The patient made a quick recovery, returning home six days after the reduction, and, from a letter received on the 22d day of February, was improving in general health rapidly.

In the early history and general treatment of this case, there are many points which strike me as worthy of a few moments' consideration. In the first place, it is almost conclusive, from the pain and hæmorrhage, that the inversion was produced by traction exerted upon the umbilical cord and placenta, when the uterus had not, and was not contracting, as it should have done after the expulsion of the child. Had the uterus thus contracted, prolonged and firm traction on the cord could not have been made, for that proper behavior of the uterus necessarily casts off the placenta. Her attendant, therefore, should have recognized, from this very retention of the placenta, that the uterus did not contract sufficiently to cast it off, and, in place of giving his attention to the placenta, he should have given it to the uterus. If the invaluable practice of having the uterus followed up by the hand had been resorted to, which was evidently not the case, it would either have stimulated the uterus to do its duty, or have shown, by its not contracting, the necessity of making it do so for no other reason than doing what misdirected efforts endeavored to accomplish. Again, had her attendant followed up the uterus during the delivery of the placenta, in the manner mentioned, he could not have failed to recognize that the organ was not contracting, but becoming inverted, and he thus would have been made aware of the danger of his procedure. Further, the character and continuance of the flooding should have told him at once that he had a non-contracted uterus; and had he then used his most ready means of ascertaining the cause of the hæmorrhage—the use of his two hands externally and internally—he would have recognized, from the absence of a uterus beneath his hand on the abdomen, and the tumor in the vagina, that the uterus was inverted. The non-observance of the ordinary precautions in a simple case of labor was undoubtedly, in this case, the cause of this patient's life being endangered by hæmorrhage, and of her suffering, during two years, from an inverted uterus.

In regard to the treatment, and its results, there are several points that seem to me to admit of a few moment's consideration. The old method of traxis certainly in this case succeeded admirably, the constricted cervix yielding gradually to the wedging effect of the fingers and the pressure of the body of the uterus. That the fingers possess a very limited power of expansion when confined thus in the vagina, I admit; but, in my opinion, this is compensated for, in a great degree, by the wedging of the uterine body between the fingers, by the upward force exerted on the palm of the hand by the arm-power of the operator. It is by this force, in my opinion, that the constricting cervical ring can be dilated sufficiently to admit of reduction of the uterine neck, and, in the case reported, I am confident that the ring was thus only dilated; as already stated, the dilating force of my fingers, *per se*, being exceedingly limited. Another point, of which slight mention is made in our leading text-books, but which in my hands worked admirably, and to which I attribute chiefly my success, is the constant use of the hot vaginal douche, preceding and succeeding each effort at reduction. The advantage of thus using hot water is self-evident, it acting in the same manner as when used in cases of rigid os, by relaxing and softening the muscular fibres of the cervix. But it was especially after efforts at reduction that it seemed to accomplish the most good, both in removing the consequent tumefaction of the uterine body, following the severe and protracted handling, as well as subduing irritation of the organ and its appendages, and thus diminishing the dangers of inflammation. Certainly, from what I saw of its effects in Mrs. R.'s case, it appears to me to afford aid of the greatest value in cases of inversion.

In conclusion, I desire to state that I essayed Courty's method of gaining a point of resistance by introducing the index and middle fingers up the rectum, but found that in such a cramped position no resistance of value could be obtained. I also gave the instrument known as White's repositor a trial, but laid it aside in a few moments, as I found it impossible to exert the pressure in the line of the uterine axis, the instrument carrying the uteri toward the promontory of the sacrum. In justice to Prof. White, I must state here that this was owing to a faulty construction of the instrument, its shaft being too straight, and its rubber cups too yielding.

Finally, I desire to express my indebtedness for invaluable assistance to my friends Drs. H. D. Nicoll, J. C. Jay, Jr., and Jeffrey Bourke, to them certainly belongs a large share of the success attending my efforts.—*New York Medical Journal.*

